# Prevalence of COVID-19 outcomes in patients referred to opioid agonist treatment centers

**DOI:** 10.3389/fphar.2023.1105176

**Published:** 2023-03-23

**Authors:** Samira Khani, Javad Tafaroji, Mehdi Yaghoubi, Mohammad Javad Emami Kazemabad, Seyed Amir Hejazi

**Affiliations:** ^1^ Neuroscience Research Center, Qom University of Medical Sciences, Qom, Iran; ^2^ Pediatric Medicine Research Center, Qom University of Medical Sciences, Qom, Iran; ^3^ Cellular and Molecular Research Center, Qom University of Medical Sciences, Qom, Iran; ^4^ Student Research Committee, School of Medicine, Qom University of Medical Sciences, Qom, Iran

**Keywords:** COVID-19, opioid, immunodulation, opioid agonist, methadone maintenance program

## Abstract

**Background:** Coronavirus disease (COVID-19) is a mild to severe infectious respiratory illness caused by the SARS-CoV-2 virus. Based on the numerous pieces of evidence regarding the role of opioids in immune function, viral replication, and virus-mediated pathology, we decided to assess the incidence and severity of COVID-19 outcomes in people undergoing opioid maintenance treatment.

**Methods:** This is a prospective, descriptive, multi-center study that included 452 patients undergoing maintenance treatment in opioid agonist treatment (OAT) clinics in different cities of Iran. Demographic information, underlying disease, history of maintenance treatment, type of drug used, history of addiction, smoking, and the kind of substance abused, were recorded. A physician evaluated the COVID-19 symptoms, and the severity of the disease was defined based on the number of observed symptoms.

**Results:** The results have not shown any significant difference in the severity of COVID-19 symptoms in different nationalities, gender, and treatment groups. Furthermore, the history of drug abuse, including time and type of abuse and smoking, has not indicated any significant association with the occurrence of symptoms. Only the severity of COVID-19 in the mentioned cities (first and second follow-up: *p* < 0.001) and individuals with a history of underlying disease (first follow-up: *p* = 0.020; second follow-up: *p* = 0.043) were significantly different.

**Conclusion:** Our results have demonstrated that the severity of symptoms in people with the underlying disease was significantly higher than in others. But there is no association between sex, race, treatment groups, and abuse history with the severity of COVID-19 symptoms in methadone maintenance treatment (MMT) patients.

## Introduction

Opioids are endogenous peptides (β-endorphin, encephalin, and met-encephalin) or exogenous substances (e.g., morphine, methadone, etc.) that act through activating opioid receptors known as µ, κ, and *δ* (Feng et al., 2012). Opioid receptors are G protein-linked receptors located in the nervous system, kidney, spleen, liver, intestine, and lung. Also, opioid receptors are distributed in the immune cells such as lymphocytes, macrophages, neutrophils, and monocytes (Wang, 2019). Recent studies have shown that opioids have immunomodulatory and anti-inflammatory effects by influencing immune cell function. Effects of opioids on immune functions include downregulation of natural killer cells and T cell activities, depression of phagocytic activity of neutrophils and macrophages; affecting cytokine receptor expression (Finley et al., 2008), diminishing the formation of antibodies, cytokines, and other inflammatory mediators (Eisenstein, 2019).

The alkaloid of morphinane in opioids, through lysosomotropic properties, inhibits viral uncoating and the entrance of coronavirus (Cismaru et al., 2021). Moreover, opioids are effective in palliating dyspnea and limiting hyperventilation by decreasing respiratory drive and corollary discharge, altering the activity of lung opioid receptors, and having anxiolytic effects (Mahler, 2013).

COVID-19 is an atypical respiratory disease caused by severe acute respiratory syndrome coronavirus-2 (SARS-CoV-2). Dysregulated inflammatory response due to hypercytokinemia, has a pivotal role in severe COVID-19. Lymphopenia, lymphocyte dysfunction, granulocyte and monocyte abnormalities, high cytokine levels, and increased total antibodies are present in COVID-19 patients (Yang et al., 2020).

Two major pathways of COVID-19 transmission to people are respiratory droplets and face-to-face contact. 14 days is regarded as a typical incubation period for COVID-19 (Rezaee et al., 2021). The real-time reverse-transcriptase-polymerase chain reduction (RT-PCR) method is used for the purpose of the COVID-19 final diagnosis (Corman et al., 2020; Rubin et al., 2020). Headache, loss of taste or smell, diarrhea, nausea, fatigue, etc., are some of the most common manifestations of this infectious disease (Chen et al., 2020; Huang et al., 2020; Lechien et al., 2020). It is reported that compared with the severe acute respiratory syndrome (SARS) coronavirus, COVID-19 has a higher level of infectivity but a lower mortality rate (Lechien et al., 2020). It seems that the mortality rate can be increased due to underlying disorders like diabetes and cancer (Nouri-Vaskeh et al., 2021).

Coronavirus is known to have four primary structural proteins: nucleocapsid proteins, envelope, membrane, and spike. The spike helps the coronavirus to enter the host cell (Li et al., 2003; Jia et al., 2005; Grass and Toole, 2015). After the first exposure, cytotoxic cells, antibodies, and interferons trigger the immune system. Alveolar infiltration of T cells, macrophages, and neutrophils occurs in the advanced levels of the disease (Khadke et al., 2020).

While attempting to provide therapies for patients with COVID-19, many treatments have been tested which often resulted in controversial consequences (Senefeld et al., 2023).

Some studies have indicated that opioid receptors have immunomodulatory effects. Therefore, it seems that opioid drugs can be effective not only in the viral infectious cycle, but also in the host’s reactions to COVID-19 by treating dyspnea, inhibiting the cytokine storm, and disrupting lysosomal acidification.

The type of virus determines the effects of the opioid system on human viral infections. The opioid system may enhance or suppress viral pathogenesis by altering host immune responses (Wang X. et al., 2005). The results of studies so far have shown that the opioid system accelerates HCV and HIV pathogenesis. On the contrary, it has a beneficial role in the outcome of respiratory viral diseases such as influenza virus and respiratory syncytial virus and could serve as effective therapeutic targets (Tahamtan et al., 2016).

Despite the use of these medications in the clinic, their role in the consequences of viral respiratory diseases has not yet been studied in detail. Due to the lack of studies related to the effectiveness of opioids in patients with COVID-19, we attempted to collect and provide some helpful information about the possible protective effects of this category of medications by evaluating patients referred to methadone maintenance treatment centers and following them up in terms of incidence, severity, and incidence of symptoms.

## Materials and methods

This is a prospective, descriptive, multi-center study that included 452 patients undergoing maintenance treatment in opioid agonist treatment (OAT) clinics in Tehran, Qom, Khorasan, Esfahan, and Kashan cities of Iran. The study protocols were approved by the ethics committee of Qom University of Medical Sciences, Qom, Iran (particular approval ID: IR.MUQ. -REC.1394.73).

Sampling was performed through the Census method. All the patients eligible for the study (452 patients) with observance of the principles of confidentiality, honesty, the confidentiality of information, etc., were entered into the study from 1 April 2017, to 20 March 2018, and followed until 11 September 2020. Demographic information (age, gender, and body mass index), underlying diseases (diabetes, hypertension, heart failure, and chronic kidney disease), history of maintenance treatments, type of drugs used, history of addiction, smoking, and the kind of substance abused, were extracted from patient statements and files.

**FIGURE 1:**
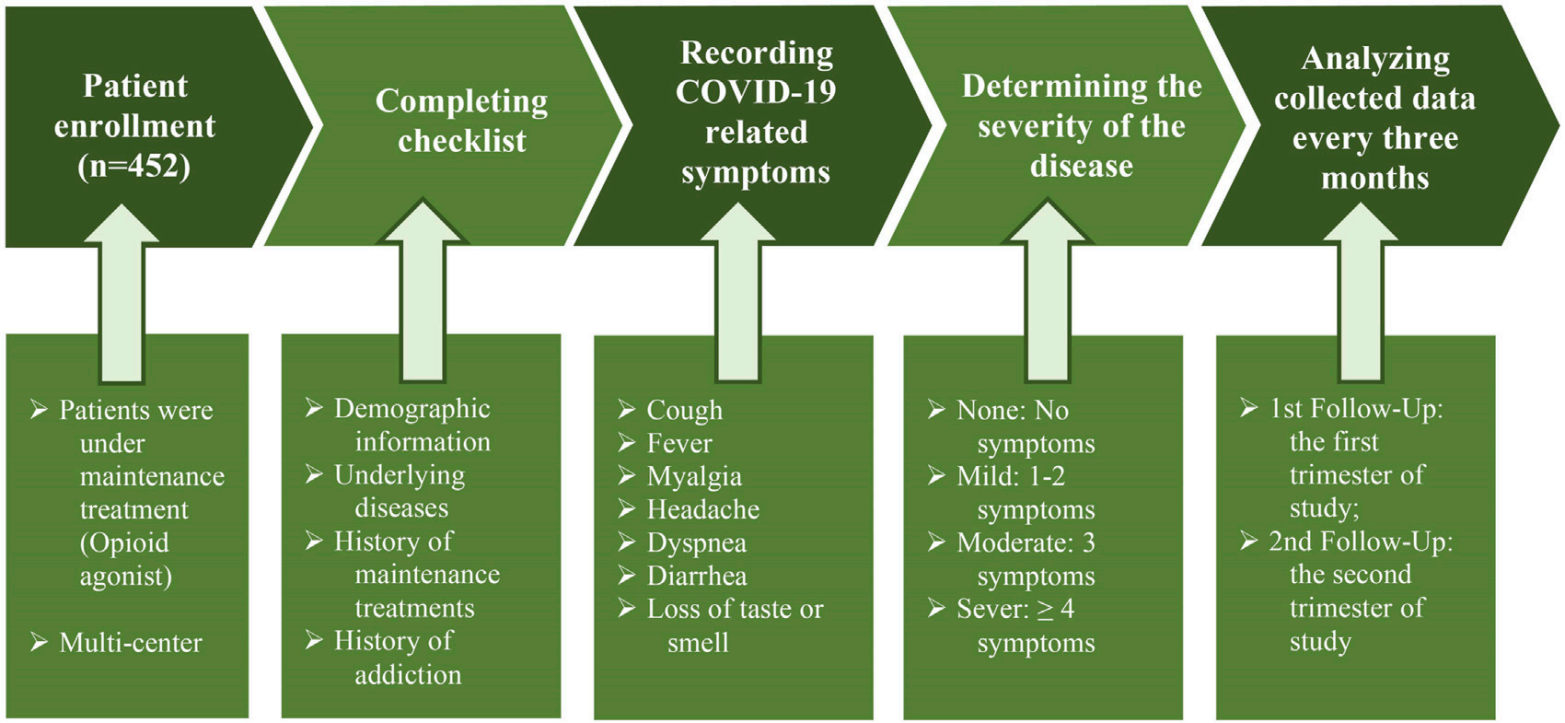
Flow diagram of participants through the study.

During the study period, patients were evaluated for COVID-related symptoms by a physician. Based on a checklist, including cough, fever, myalgia, headache, dyspnea, diarrhea, and loss of taste or smell, all the symptoms were recorded. The severity of the disease was defined based on the number of symptoms observed in the patient as follows: None: No symptoms; Mild: One to two symptoms; Moderate: Three symptoms; Severe: ≥4 symptoms. Finally, the severity of the disease was evaluated in the mentioned period. We analyzed collected data every 3 months and presented it as the first follow-up: the first trimester of study; second follow-up: the second trimester of study, in this group of patients. Furthermore, the hospitalization rate and mortality were recorded during the study (Figure 1).

## Statistical analysis

The SPSS statistical software version 25.0 (SPSS Inc., Chicago, IL, United States of America) was used for data analysis. Age, BMI, history of drug abuse, and MMT history (year), were expressed as the mean ± SD and were compared using ONEWAY ANOVA, *post hoc* Tukey test. The remaining parameters (e.g., gender, nationality, etc.), were presented as n (%), and the comparisons between different severity groups were examined by the Chi-square test. P-value <0.05 was considered statistically significant.

## Results

During the study period, 452 patients were enrolled in this research. The median age was 44.92 ± 12.06 years, and 426 (94.3%) were men. Table 1 shows the demographic characteristics of those who entered the study. The underlying disease and medication history of patients have summarized in Table 2. 77.4% of patients had not any underlying disease. Heart disease (6.6%) and hypertension (10.6%) were more common among patients. 93.8% of patients had a history of drug abuse for more than 1 year, and opium addiction is more common (82.3%) in these patients. 75.2% of patients have used methadone as maintenance therapy.

**TABLE 1:** Demographic information of patients referred to MMT centers. (n = 452).

Parameters	(n = 452)
Age, mean ± SD	44.92 ± 12.06
Gender, n (%)	
Female	26 (5.7%)
Male	426 (94.3%)
BMI, mean ± SD	23.52 ± 2.65
Nationality, n (%)	
Iran	429 (94.9%)
Afghanistan	19 (4.2%)
Arab	4 (0.9%)
City, n (%)	
Qom	160 (35.4%)
Tehran	76 (16.8%)
Mashhad	71 (15.7%)
Esfahan	71 (15.7%)
Kashan	74 (16.4%)

**TABLE 2:** Disease and medication history of patients referred to MMT centers. (n = 452).

History	n = 452
Underlying diseases, n (%)	
Any disease	350 (77.4)
Heart	30 (6.6)
Hypertension	48 (10.6)
Kidney	13 (2.9)
Diabetes	9 (2)
Rheumatoid	2 (0.4)
Drug abuse history, n (%)	
<1 (year)	28 (6.2)
1–5	141 (31.2)
6–10	99 (21.9)
11–15	64 (14.2)
16–20	85 (18.8)
>20	35 (7.7)
Drug abuse, n (%)	
Opium	372 (82.3)
Marijuana	9 (2)
Methamphetamines	16 (3.5)
Crack	1 (0.2)
Multidrug	54 (11.9)
MMT history, n (%)	
1–3 (year)	193 (42.7)
4–6	164 (36.3)
7–9	74 (16.4)
10–12	18 (4)
13–15	3 (0.7)
MMT drug, n (%)	
Opium	28 (6.2)
Methadone	340 (75.2)
Buprenorphine	84 (18.6)

**FIGURE 2:**
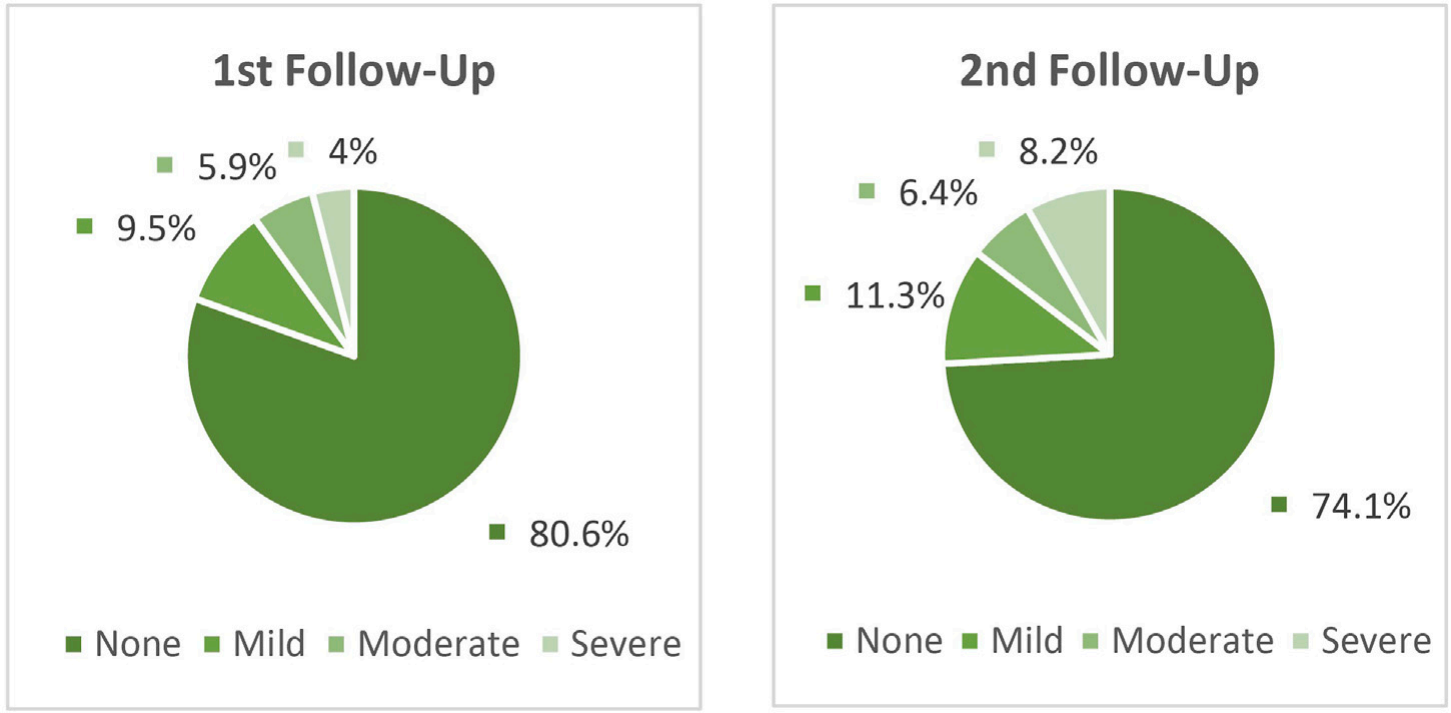
Symptoms severity of patients referred to MMT centers at first and second follow-ups (n = 452); None, no symptom; Mild, one to two symptoms; Moderate, three symptoms; Severe, ≥4 symptoms.

**FIGURE 3:**
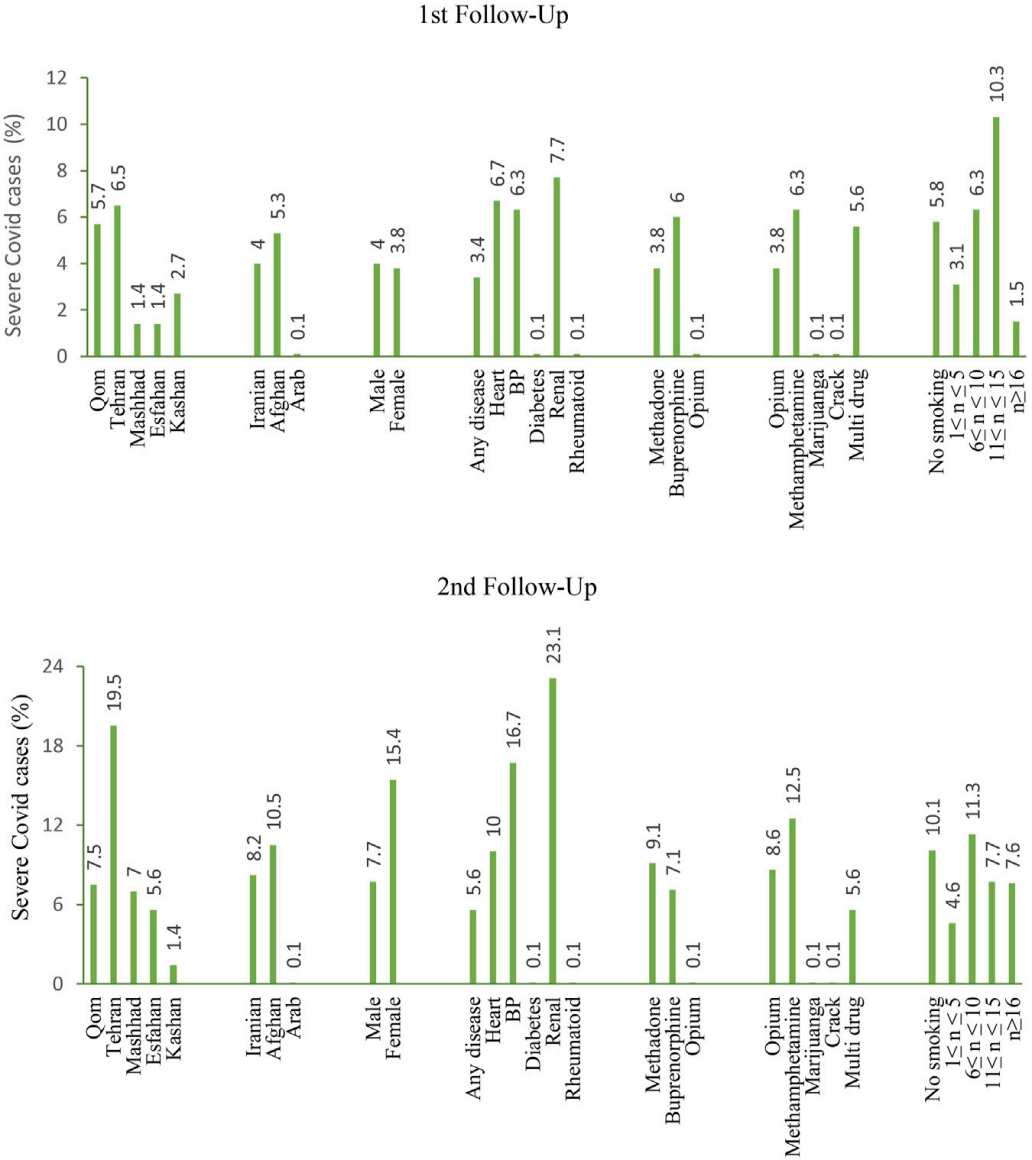
Percentage of patients with severe COVID-19 (≥4 symptoms) referred to MMT centers in the first and second follow-ups.

The prevalence of COVID-19 symptoms in the second trimester of the study period (second follow-up) was higher among patients than in the first trimester (first follow-up) (Table 3). 80.6% and 74.1% of patients in first and second follow-up respectively, have not shown any symptoms (Figure 2). The results also have not shown any significant difference in the severity of COVID-19 symptoms in different nationalities (first follow-up: *p* = 0.241; second follow-up: *p* = 0.376), gender (first follow-up: *p* = 0.972; second follow-up: *p* = 0.503), and treatment groups (first follow-up: *p* = 0.565; second follow-up: *p* = 0.444). Furthermore, no statistically significant association was observed between the history of drug abuse (first follow-up: *p* = 0.671; second follow-up: *p* = 0.182) and smoking (first follow-up: *p* = 0.623; second follow-up: *p* = 0.788) with the occurrence of symptoms. Only the prevalence of symptoms in the mentioned cities (first and second follow-up: *p* < 0.001) and individuals with a history of underlying disease (first follow-up: *p* = 0.020; second follow-up: *p* = 0.043) were significantly different (Figure 3).

**TABLE 3:** Clinical symptoms of patients referred to MMT centers. (n = 452).

Symptom	First follow-up	Second follow-up
None	364 (80.5)	335 (74.1)
Cough, n (%)	23 (5.1)	37 (8.2)
Fever, n (%)	37 (8.2)	54 (11.9)
Myalgia, n (%)	34 (7.5)	53 (11.7)
Headache, n (%)	39 (8.6)	53 (11.7)
Dyspnea, n (%)	23 (5.1)	42 (9.3)
Diarrhea, n (%)	46 (10.2)	59 (13.1)
Loss of taste or smell, n (%)	26 (5.8)	49 (10.8)

During a 6-month study duration, 4 (0.88%) death were recorded among the 452 patients referred to MMT clinics. 12 (2.65%) patients were hospitalized for COVID, as well.

## Discussion

The debate on whether opioids could help to improve or prevent COVID-19 has arisen since the beginning of the outbreak of this pandemic. So, we decided to investigate the incidence of symptoms and hospitalization in patients undergoing opioid maintenance therapy. Opioid agonist medications, including methadone, buprenorphine, and opium, have been administered at a specialized addiction clinic in Iran to treat opioid use disorder (OUD). In this study, the incidence rate of clinical symptom due to COVID-9 in patients undergoing different maintenance therapy treatment have not shown any significant difference. Also, other factors such as sex, body mass index (BMI), race, smoking, and abuse history have not been associated with COVID-19 symptom occurrence.

Salimi et al. have shown that opioid receptor signaling can control the severity of respiratory disease by influencing syncytial virus replication. Other clinical and experimental studies also have indicated that opioid receptor signaling has a potentially beneficial role in the outcome of respiratory viral disease. In influenza and respiratory syncytial viruses (RSVs), opioids reduce the rate and severity of infection through anti-inflammatory and immunomodulatory functions (Salimi et al., 2013).

Cismaru et al. (2021) previously reported that none of the opioid medications are superior to others in treating dyspnea. So, oxycodone, hydromorphone fentanyl dihydrocodeine, diamorphine, and oral or parenteral morphine can be used for this purpose.

Matsunaga M showed genetic differences are effective in expressing opioid receptors in immune function and secretion of proinflammatory factors IL6, IL8, and TNF-α (Matsunaga et al., 2009).

Maher et al. (2019) showed that opioid peptides have suppressive effects on the cytotoxic function of natural killer cells. Stimulating opioid receptors reduce mucus secretion in the respiratory system (Krajnik et al., 2014). They also play a significant role in immune system activity, including antibody production, lymphocyte proliferation, and modulation of cytokine production. Furthermore, Opioids suppress IL6 signaling pathways (Eisenstein, 2019).

De Wilde et al. (2014) demonstrated loperamide as an opioid receptor agonist inhibits MERS-CoV, SARS coronavirus, and human coronavirus 229E replication.

On the other hand, El-Hage et al. (2011) reported that morphine is involved in the pathogenesis of hepatitis C virus-infected hepatocytes by impairing cytokine expression and altering the production of reactive oxygen species and nitric oxide. Furthermore, morphine enhances the severity of primary herpes virus infection (Mojadadi et al., 2009) and secondary bacterial pneumonia lung infection (Wang J. et al., 2005).

Since using opioids is known to have immunosuppressive effects, infection due to the 2019 novel coronavirus disease has been one of the major concerns in people using opioids during the pandemic. It is reported that there is a considerable association between ICU admission and using opioids in patients with COVID-19. It is also worth mentioning that compared to non-users, higher mortality is recorded among opioid users (Ao et al., 2022).

In a study regarding long-term opioid therapy in patients, it was stated that due to compromised respiratory and immune function, susceptibility to COVID-19 infection increases. It was also reported that the risk of mortality and morbidity increases in these patients (Tuan et al., 2021).

Therefore, all aspects should be weighed before using opioids to treat an inflammatory viral respiratory infection, and caution is required.

Numerous studies have demonstrated the anti-inflammatory and immunomodulatory effects of opioids and the presence of opioid peptides in immune cells to reduce inflammatory hyperalgesia (Machelska and Celik, 2020).

It has also been shown that immune factors are significantly reduced after exposure of consumers (drug users, healthy human subjects, and animal models) to opioid compounds (Roy et al., 2011). However, this immunosuppression efficacy cannot be generalized to all opioid compounds. Just morphine, fentanyl, and remifentanil have immunosuppressive effects, and other compounds such as tramadol and buprenorphine have neutral impacts on the immunity system. Animal studies have shown a suppressive effect of methadone, but clinical studies as maintenance therapy in heroin-addicted patients have indicated immune function restoration (Franchi et al., 2019).

Ghafari et al. (2021) have found that Iran has a significantly high death rate in the spring and summer of 2020 due to the uncontrolled transmission of the virus.

Concerning, the high estimated level of exposure in Qom (57% exposed) based on previous data compared to other cities in this study, it is expected to have a higher mortality rate during the time of the crisis, which results do not show such a finding compared to Tehran with 22% population-level exposure to the virus (Ghafari et al., 2021). However, Small sample sizes pose challenges to any statistical analyses.

Many studies have demonstrated that older age, male sex, race (mainly Black, Hispanic, and South Asian), and underlying comorbidities have been associated with disease severity or death of COVID-19 patients (Attaway et al., 2021)*.* Our results also have shown that the severity of symptoms in people with the underlying diseases was significantly different from others. But sex and race did not have significant effects on the severity of COVID-19 symptoms in MMT patients.

The limitation of this study was the lack of a control group (do not consume any opioid compounds) to investigate the relationship between opioid use and COVID-19 outcomes. Therefore, large-scale studies in different ethnic and geographical groups are needed to evaluate the relationship between opioids and survival in COVID-19 and clarify the effects of opioids on the clinical prognosis of COVID-19 patients. Therefore, it is suggested that studies be conducted to investigate how opioid use may impact immunity in the lungs and alter viral replication and virus-mediated pathology in COVID-19. We also did not have complete information on the mortality rate of patients in light centers in the same period of previous years to estimate excess mortality associated with COVID-19. Also, we only had access to the data of a limited number of MMT centers related to COVID-19 in different parts of Iran. Another limitation of this study was the lack of accompanying patients to complete laboratory tests and chest CTs. Therefore, the complete laboratory data was not available to the researchers for analysis.

Finally, more research needs to be done to determine if opioid administration can be helpful in viral diseases, specially COVID-19, or not. Furthermore, opioid addiction and misuse should be considered, and the potential benefits and harms during treatment should be weighed.

## Conclusion

Considering that the anti-inflammatory and immunomodulatory effects of opioids have been demonstrated in numerous studies, we decided to investigate the incidence of symptoms and hospitalization in patients undergoing opioid maintenance therapy. Our results have indicated that the severity of symptoms in people with the underlying disease was significantly higher than in other patients who did not have any underlying disease. But sex, race, treatment groups, and abuse history did not have any significant association with the severity of COVID-19 symptoms in methadone maintenance treatment (MMT) patients.

## Data Availability

The raw data supporting the conclusion of this article will be made available by the authors, without undue reservation.
